# TREM2^+^ macrophages: a key role in disease development

**DOI:** 10.3389/fimmu.2025.1550893

**Published:** 2025-04-02

**Authors:** Xinxin Wang, Yunhan Wang, Lei Yang, Yi Zhang, Li Yang

**Affiliations:** ^1^ Biotherapy Center and Cancer Center, The First Affiliated Hospital of Zhengzhou University, Zhengzhou, China; ^2^ School of Life Sciences, Zhengzhou University, Zhengzhou, China; ^3^ State Key Laboratory of Esophageal Cancer Prevention & Treatment, Zhengzhou, China; ^4^ Tianjian Laboratory of Advanced Biomedical Sciences, Academy of Medical Sciences, Zhengzhou University, Zhengzhou, China; ^5^ School of Public Health, Zhengzhou University, Zhengzhou, China

**Keywords:** TREM2, macrophages, signaling pathway, disease, targeted therapy

## Abstract

Triggering receptors expressed on myeloid cells 2 (TREM2), an immune receptor expressed on myeloid cells, has garnered considerable attention in recent years due to its role in unique signaling pathways and diverse biological functions, including phagocytosis, lipid metabolism, cell survival, and inflammatory responses. Although TREM2 is expressed in various cell types, such as macrophages, dendritic cells (DCs), osteoclasts, and others, where it exhibits context-dependent functional characteristics, it is mainly expressed in macrophages. Notably, TREM2 is implicated in the development and progression of multiple diseases, playing dual and often opposing roles in noncancerous diseases and cancers. This review aims to highlight the pivotal role of TREM2 in macrophages and immune-related diseases, elucidate its underlying mechanisms of action, explore its potential as a clinical diagnostic and prognostic marker, and propose therapeutic strategies targeting TREM2 based on current clinical trial data, providing comprehensive guidance and references for clinical practice.

## 1 Introduction

In complex biological systems, immune receptors play vital roles in maintaining homeostasis and responding to external challenges. The immune system acts as the first line of defense against invading pathogens and preserves internal stability. Recently, as research on triggering receptors expressed on myeloid cells 2 (TREM2), has advanced, its central role in regulating immune responses, maintaining tissue homeostasis, and contributing to disease processes has gained recognition. TREM2 is a single-pass transmembrane receptor of the immunoglobulin superfamily. According to the NCBI Gene database (https://www.ncbi.nlm.nih.gov/gene/?term=TREM2), the TREM2 encoding gene is located on chromosome 6p21.1 of human chromosome and on mouse chromosome 17, and both contain 5 coding exons. Single-cell RNA sequencing (scRNA-seq) revealed that among other cell types, TREM2 is also expressed in specific macrophages of the human brain, lungs, adipose tissue, adrenal gland, and placenta (1); however, its precise functions in these tissues remain to be further elucidated. Furthermore, the expression of TREM2 in the central nervous system (CNS) exhibits significant regional differences. It is most strongly expressed in the basal ganglia, corpus callosum, medulla oblongata, and spinal cord (2). Specifically, in white matter, the expression level of TREM2 is 4.96 times higher than that in the cerebellum (p = 6.63 × 10^-^³³). This may be related to the higher density or activity of microglia in white matter. Additionally, there is no significant gender difference in TREM2 expression in any brain region (3). Chertoff M et al. observed the expression of TREM2 protein in the brains of C57BL/6 mice from postnatal day 1 to day 14 and found that the expression of TREM2 in microglia exhibited region- and age-dependent downregulation. In the gray matter, TREM2 expression became almost undetectable after postnatal day 5, while in the white matter, particularly in the corpus callosum, TREM2 expression persisted until postnatal day 10. This indicates that TREM2 expression lasts longer in white matter regions compared to gray matter regions (4).

We focused on how TREM2 interacts with various ligands through its unique V-type immunoglobulin domain, thereby mediating the activation of spleen tyrosine kinase (Syk) and Phosphatidylinositol 3-kinase (PI3K) and triggering a series of intracellular signaling events (5–7). These events regulate phagocytosis, metabolism, inflammatory responses, and the survival and proliferation of macrophages. Notably, TREM2 has been identified as a novel anticancer target, with high expression in tumor-associated macrophages (TAMs) and minimal or no expression in macrophages within normal peripheral tissues. Therefore, inhibiting TREM2 holds promise as an effective approach of eliminating cancer cells. In summary, elucidating the biological functions and mechanisms of action of TREM2 is crucial for developing new therapeutic strategies targeting this receptor.

## 2 Role of TREM2 in various cells

Recently, the functions of TREM2 in various cell types have garnered increasing attention. Numerous studies have shown that TREM2 is predominantly expressed in macrophages, dendritic cells (DCs), and osteoclasts derived from myeloid progenitor cells. TREM2 is also expressed in myeloid-derived suppressor cells (MDSCs), neutrophils, eosinophils, natural killer (NK) cells, T cells and mesenchymal stem cells (MSCs). However, TREM2 is not expressed in human peripheral blood monocytes due to low levels of H3K4me3 and AcH3 modifications in the promoter region of *Trem2* in monocytes (3, 8–11) (**Table 1**).

**Table 1:** The function of TREM2 in various cell types.

Cell types	Functions	References
Macrophages/Microglia	Associated with the phagocytosis of microglia	(4, 15–19, 25, 55, 59, 61, 66–76)
	Associated with metabolic homeostasis of microglia	(4, 61, 66, 70, 82, 98, 122)
	Involved in innate immune response	(2, 13–16)
	Playing a protective role in CNS degenerative diseases such as AD	(16–19, 61, 66, 70, 71)
	Playing a protective role in CNS inflammatory diseases such as EAE and MS	(10, 20, 21)
	Associated with immune suppression and cancer progression	(22, 35, 47, 76, 111, 112, 118, 119, 124, 125, 127, 129, 130, 133, 134, 136–140)
	Maintaining the quiescence of hair follicle stem cell and inhibiting hair growth	(12)
	Involved in synapse elimination	(4, 15)
Alveolar macrophages	Involved in the repair of damaged lung tissue in COPD	(24)
Hepatic Kupffer cells	Involved in the elimination of Plasmodium	(27)
	Involved in the protection against liver injury	(25, 26, 28, 29, 31)
	Protecting hepatocytes from undergoing carcinogenesis	(30)
Dendritic cells	Promoting the maturation and migration of DCs	(9, 32, 33, 37)
	Inhibiting the proliferation of T cells	(35)
	Participating in immunosuppression of lung cancer	(35)
	Involved in allergic airway inflammation such as asthma	(33)
	Promoting the progression of IBD	(34)
	Involved in the protection against renal injury of the CKD	(36)
Osteoclasts	Regulating the differentiation and function of osteoclasts	(32, 38–42)
	Involved in osteoporosis	(39)
	Involved in Nasu Hakola disease	(38)
	Involved in acquired cholesteatoma-induced bone destruction	(44)
	Involved in periodontitis induced bone destruction	(45)
MDSCs	Involved in anti-tumor effects in DLBCL	(48)
Neutrophils	Promoting the growth and metastasis of prostate cancer	(49)
NK cells	Regulates the development and differentiation of NK cells	(50)
	Involved in anti-tumor effects in melanoma	(50)
T cells	Activating the CD3/ZAP70/STAT1 signaling pathway in SARS-CoV-2 patients	(51)
Eosinophils	Regulating the differentiation and maturation of eosinophils	(52)
	Involved in the repair of damaged muscle tissue and non pulmonary sarcoidosis	(52)
Mesenchymal stem cells	Negatively regulating inflammatory response in MSCs	(53)

Under physiological conditions, TREM2^+^ macrophages secrete OSM to activate the JAK-STAT5 signaling pathway, maintaining the quiescence of hair follicle stem cells, which is crucial for the long-term health and stability of hair follicles. However, if hair follicle stem cells remain in a prolonged quiescent state and fail to be activated, it may prevent hair follicles from entering the growth phase, leading to hair loss. Targeting TREM2^+^ macrophages or the OSM signaling pathway may provide a novel strategy for treating hair loss (12). In the central nervous system (CNS), TREM2 is primarily expressed in microglia, where it plays a crucial role in the innate immune responses (2, 13–16). By engaging the DNAX-activating protein 12 kDa (DAP12)-coupled immunoreceptor tyrosine-based activation motif (ITAM) signaling, TREM2 activates microglia to clear cellular debris and synaptic connections, which are crucial for tissue regeneration and repair, as well as for maintaining brain homeostasis (4, 15). Under various pathological conditions, such as infection, inflammation, and cancer, the expression and function of TREM2 often undergo significant changes. ScRNA-seq revealed a group of disease-associated microglia (DAM) whose activation is considered a protective response (16, 17). During Alzheimer’s disease (AD) progression, the TREM2-TYROBP complex is involved in regulating the activation of DAM, that expresses high levels of phagocytic and lipid metabolism-related genes, suggesting its potential role in clearing amyloid-β (Aβ) plaques (16, 17). The absence of TREM2 leads to a decreased ability of microglia to clear Aβ plaques and affects their transition to the DAM phenotype, which may exacerbate the pathological progression of AD (18). Upregulating TREM2 expression can reprogram the responsiveness of microglia, reduce neuropathological features in AD mouse models, and improve cognitive function (19). In addition to the aforementioned CNS degenerative diseases such as AD, TREM2-positive microglia also play a protective role in CNS inflammatory diseases such as experimental autoimmune encephalomyelitis (EAE) (20, 21) and multiple sclerosis (MS) (10). In human tumors, TREM2 is mainly expressed in tumor-associated macrophages (TAMs) and is often associated with immune suppression and poor prognosis (22). Additionally, other types of tissue-resident macrophages, such as alveolar macrophages (23, 24) and hepatic Kupffer cells (KCs) (25–31) also highly express TREM2.

DCs play a central role in antigen presentation and T-cell activation. Studies have reported that TREM2 receptors are expressed in human cerebrospinal fluid (CSF) monocytes, while they are not expressed in peripheral blood monocytes. However, during the differentiation of monocytes into DCs, the expression of TREM2 significantly increases. This suggests that the CNS microenvironment has a certain influence on the phenotype of monocytes (3, 8–11). Under the stimulation of GM-CSF and IL-4, TREM2 is expressed in monocyte-derived DCs (32). TREM2-positive DCs exhibit higher levels of CCR7 and CD86 expression, which suggests that TREM2 may play a role in the maturation and migration of DCs (9, 33). Moreover, TREM2-positive DCs promote the activation of Th2 and Th17 cells, thereby further exacerbating airway hyperresponsiveness and contributing to the development of asthma (33). In the inflamed intestinal mucosa of inflammatory bowel disease (IBD) patients, TREM2 is mainly expressed on the surface of mature CD11c^+^ DCs and promotes the release of pro-inflammatory cytokines, such as TNF-α, IL-6, IL-1β, and IL-12p70, as well as the activation of T cells, thereby contributing to the progression of IBD (34). In the lung cancer mouse model, the expression of TREM2 in pulmonary CD11c+ DCs is increased. These TREM2^+^ DCs exhibit a phenotype characterized by low expression of CD80, CD86, and MHC II, along with decreased secretion of IL-12 and increased production of IL-10. Furthermore, they demonstrate an enhanced capacity to suppress T cell proliferation. *In vivo* experiments have observed that adoptive transfer of TREM2^+^ DCs can accelerate tumor growth in lung cancer-bearing mice but does not significantly affect their survival (35). In the unilateral ureteral obstruction (UUO) mouse model, TREM2 is highly expressed in DCs and exerts a protective role in chronic kidney disease (CKD) by regulating the production of NO to inhibit the differentiation of Th17 cells and the production of IL-17, thereby alleviating renal inflammation and fibrosis (36). A novel synthetic sulfolipid, Sulfavant A, binds to TREM2, enhancing DC migration and activation (37). Osteoclasts, derived from the monocyte/macrophage lineage, are the only cells capable of bone resorption, and TREM2-DAP12 complex regulates the osteoclast formation, fusion, and bone resorption. The knockout of TREM2 or DAP12 delays osteoclast differentiation and defects in actin cytoskeleton rearrangement (32, 38–42). The accumulation of numerous immature osteoclasts impairs bone resorption, that leads to osteoporosis (39) and, in severe cases, polycystic bone lesions, such as Nasu-Hakola disease (NHD), also known as Polycystic Lipomembranous Osteodysplasia with Sclerosing Leukoencephalopathy (PLOSL) (43). In addition, TREM2 interacts with other receptors or molecules, playing a role in various pathological processes. DAP12, through its interaction with CSF1R, influences cytoskeletal reorganization and cell cycle progression (40, 42). TREM2 amplifies the inflammatory response of the TLR4 signaling pathway, promoting the secretion of MMP-2 and MMP-8 and the excessive activation of osteoclasts, thereby facilitating bone destruction induced by acquired cholesteatoma (44). Furthermore, the TREM2-DAP12 complex mediates a Syk-dependent amplification of reactive oxygen species (ROS), enhancing osteoclast differentiation and activation, which contributes to alveolar bone destruction in periodontitis (45). MDSCs originate from immature myeloid cells (IMCs) in the bone marrow. Studies have shown that TREM2 is primarily expressed in monocytic MDSCs (Mo-MDSCs) and to a lesser extent in granulocyte-like MDSCs (PMN-MDSCs) (46, 47). In diffuse large B-cell lymphoma (DLBCL), the expression of TREM2 on M-MDSCs has been found to be associated with immunosuppressive functions, potentially by upregulating the expression of Arginase 1 (ARG1) to inhibit the proliferation of CD8^+^ T cells, thereby promoting tumor growth. In light of this, the expression level of TREM2 on circulating M-MDSCs may serve as a novel immunotherapeutic target. By targeting TREM2, it is possible to attenuate the immunosuppressive capacity of M-MDSCs, enhance the body’s anti-tumor immune response, and ultimately improve the prognosis of DLBCL patients (48). Neutrophils and NK cells are the key components of the innate immune system, and prior studies have shown that prostate cancer cells induce senescence in TREM2^+^ immunosuppressive neutrophils by secreting APOE. Senescent neutrophils exhibit a stronger immunosuppressive effect than that of typical immunosuppressive neutrophils, that facilitates tumor growth and metastasis. Genetic inactivation of the *Apoe* or *Trem2* genes or the use of the histone deacetylase inhibitor romidepsin to specifically eliminate senescent neutrophils has been shown to effectively slow the progression of prostate cancer in mouse models (49). As for NK cells, Lee et al. were the first to demonstrate that TREM2 is expressed in CD3^−^CD122^+^NK1.1^+^ precursor NK (pNK) cells, and that TREM2 regulates the development and differentiation of NK cells through the PI3K signaling pathway. The tumor volume in TREM2-overexpressing transgenic (TREM2-TG) B16F10 melanoma mice was significantly smaller than that in WT mice, suggesting that TREM2 activation can enhance the killing of melanoma cells by promoting the development and function of NK cells (50). T cells, as central components of the adaptive immune system, play a critical role in the host defense against infections. In patients infected with SARS-CoV-2, the expression of TREM2 is significantly upregulated in both CD4^+^ and CD8^+^ T cells, where it activates the CD3/ZAP70/STAT1 signaling pathway, thereby promoting a TH1-type immune response. This upregulation of TREM2 in T cells is positively correlated with the severity of COVID-19. In contrast, TREM-2 expression is nearly undetectable in T cells from healthy individuals (51). Eosinophils play important roles in allergic reactions and parasitic infections, and TREM2 is crucial for their differentiation and maturation. There is a significant positive correlation between the percentage of TREM2^+^ eosinophils and body mass index (BMI) (R² = 0.57, p = 0.012) and total white blood cell counts (R² = 0.59, p = 0.012) in humans, which suggests that TREM2 can serve as a potential biomarker for assessing the inflammatory status and metabolic condition of the body (52). MSCs are pluripotent stem cells with varying differentiation potential, and TREM2 has also been reported to play an important regulatory role in MSC differentiation, particularly in the negative regulation of LPS-mediated inflammatory responses (53, 54).

In summary, although TREM2 is expressed in various immune cells, current researches reported that TREM2 is mainly expressed in macrophages (1, 55). From the Human Protein Atlas website (https://www.proteinatlas.org/ENSG00000095970-TREM2/single+cell) and the Human Cell Landscape (HCL) website (https://bis.zju.edu.cn/HCL/landscape.html), we can concluded that TREM2 is particularly highly expressed in macrophages across different cell types. Therefore, this review mainly examines the function of TREM2 in macrophages and explores its association with immune-related diseases.

## 3 The TREM2 signaling pathway

**Figure 1:**
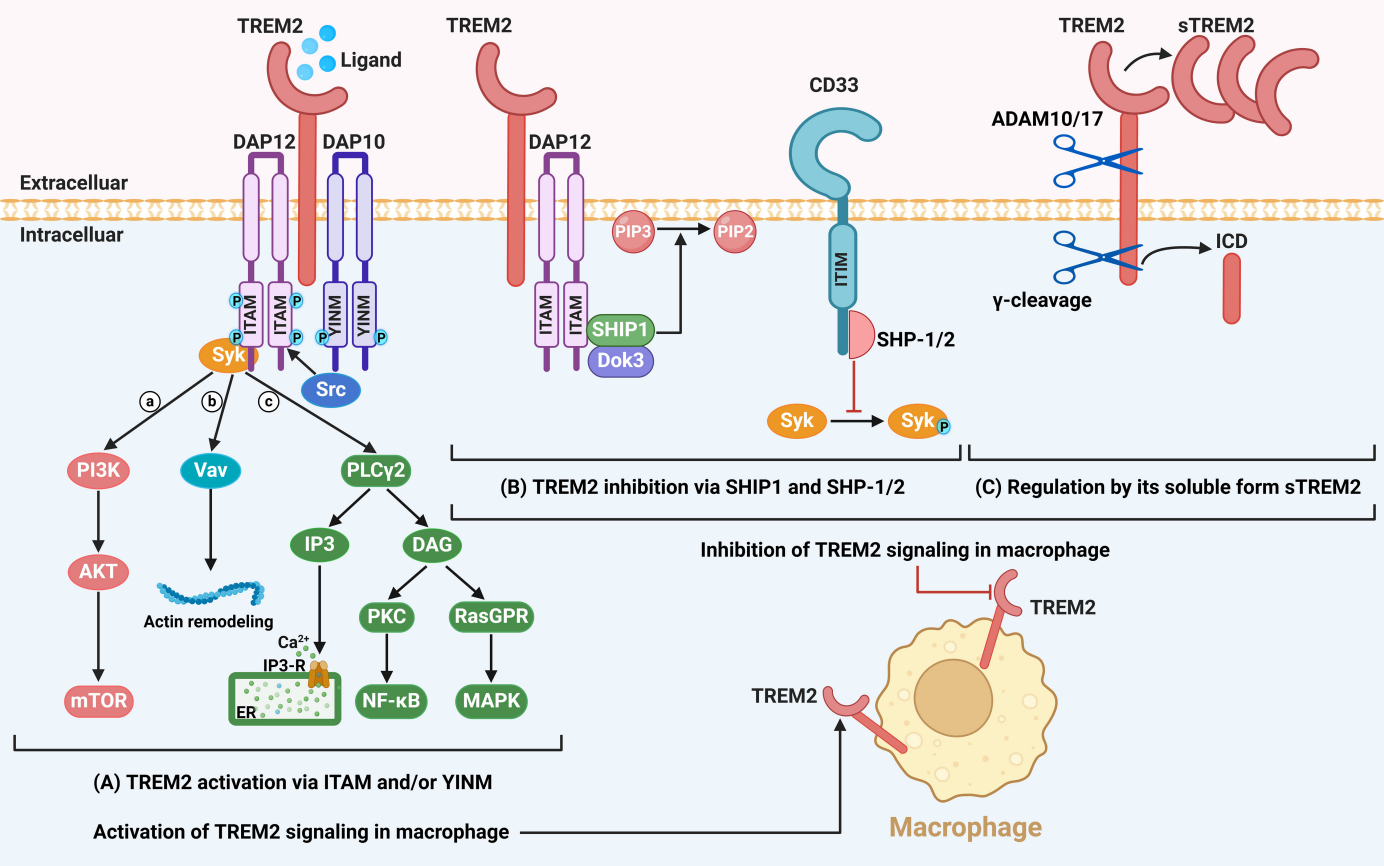
The TREM2 signaling pathway. **(A)** TREM2 consists of an extracellular domain containing a V-type immunoglobulin-like domain, a transmembrane region, and a short cytoplasmic domain lacking intrinsic signaling motifs. TREM2 functions by forming a heterodimeric complex with the adaptor proteins DAP10 or DAP12. Upon ligand binding, the immunoreceptor tyrosine-based activation motif (ITAM) within DAP12 undergoes tyrosine phosphorylation, which recruits and activates the tyrosine kinase Syk. This subsequently initiates a cascade of downstream signaling events, including the activation of the PI3K/AKT-mTOR pathway, actin remodeling, Ca^2+^ mobilization, and the activation of the NF-κB and MAPK signaling pathways. **(B)** The ITAM motif of DAP12 can also interact with the inhibitory proteins SHIP1, which mediates the degradation of PIP3 to PIP2, thereby attenuating TREM2 signaling. Additionally, CD33 can recruit phosphatases SHP1 or SHP2 to inhibit Syk phosphorylation, providing a cross-regulatory mechanism to suppress TREM2 signaling. **(C)** Furthermore, TREM2 signaling is modulated by proteolytic processing. The extracellular domain of TREM2 is cleaved by ADAM10/17, releasing soluble TREM2 (sTREM2), while the intracellular domain (ICD) of TREM2 is cleaved by γ-secretase, generating TREM2 ICD. Both processes contribute to the downregulation of TREM2 signaling activity. Image created with BioRender.com.

TREM2 consists of an extracellular region, a transmembrane region, and a short cytoplasmic region (5–7, 56–59) (**Figure 1A**). The extracellular region of TREM2 contains a V-type immunoglobulin (IgV) domain and a long stalk region. The IgV domain, located at the N-terminus, contains positively charged arginine residues, which enable it to interact with negatively charged ligands to initiate signal transduction. The IgV domain is connected to the transmembrane region via the long stalk region. The transmembrane region of TREM2 is primarily composed of helical structures, which anchor it to the phospholipid bilayer of the cell membrane through hydrophobic interactions. The intracellular C-terminus domain of TREM2 lacks signaling motifs. Therefore, TREM2 primarily mediates downstream signaling through electrostatic interactions between the basic lysine residues in its transmembrane domain and the acidic aspartic acid residues in the transmembrane domains of DNAX-activating protein of 12 kDa (DAP12) and DNAX-activating protein of 10 kDa (DAP10) (5–7, 56–59).

DAP12 is also known as tyrosine kinase-binding protein (TYROBP) (6). Upon ligand recognition, the ITAM within DAP12 is phosphorylated on two tyrosine residues by Src family kinases, that provides a docking site for recruitment and activation of Syk through its phosphotyrosine-interacting Src homology 2 (SH2) domain (5, 59, 60). Syk activation leads to the phosphorylation of multiple downstream signaling molecules, such as PI3K (56). Notably, the signaling function of DAP10 and DAP12 are distinct. The tyrosine-isoleucine-asparagine-methionine (YINM) motif within the cytoplasmic domain of DAP10 allows the recruitment and binding of PI3K onto the cell membrane after tyrosine phosphorylation, that in turn converts phosphatidylinositol 4,5-bisphosphate (PIP_2_) to phosphatidylinositol 3,4,5-trisphosphate (PIP_3_) and subsequently activates PI3K-AKT-mTOR signaling (5, 7, 56, 59). Additionally, PIP_3_ generation leads to the recruitment of linker for activation of T cells (LAT). As a key molecule in signal transduction, LAT effectively recruits the SOS1-Grb2-Vav guanine nucleotide exchange factor 1 (Vav1) complex via SOS1 and phospholipase Cγ2 (PLCγ2). Once activated, Vav1 promotes actin remodeling and regulates cytoskeletal reorganization, which is essential for the phagocytic function of microglia and their response to pathological injuries (59). On the other hand, LAT also recruits signaling adaptors such as PLCγ2. PLCγ2 then catalyzes the hydrolysis of PIP_2_ into diacylglycerol (DAG) and inositol 1,4,5-trisphosphate (IP_3_). IP_3_ then binds to IP_3_-gated calcium channels in the endoplasmic reticulum, leading to the release of Ca^2+^ from the endoplasmic reticulum into the cytoplasm, thereby increasing intracellular Ca^2+^ concentration. DAG further activates protein kinase C (PKC) and Ras guanyl nucleotide releasing proteins (RasGRPs), which then activate the NF-κB and mitogen-activated protein kinase (MAPK) pathways (5, 7, 61). The activation of PLCγ2 ultimately supports multiple biological processes, including lipid metabolism, phagocytosis, and cell survival. Furthermore, in Alzheimer’s disease, PLCγ2 may exert protective effects by facilitating the clearance of dead cells and pathological aggregates by microglia (61).

Conversely, SH2 domain-containing inositol phosphatase-1 (SHIP1) negatively regulates TREM2 signaling. SHIP1 directly binds to DAP12 through its SH2 domain and dephosphorylates PIP_3_ back to PIP_2_, thereby preventing the binding of PI3K to DAP12 and inhibiting cellular activation (62) (**Figure 1B**). Also, SH2 domain-containing protein tyrosine phosphatase 1 (SHP-1) and SHP-2 can bind to CD33 (also called “Siglec-3”) containing ITIM motif to inhibit DAP12-mediated Syk phosphorylation (63). In summary, both SHIP1 and SHP-1/2 inhibit the TREM2/DAP12 pathway in different ways. In addition to its membrane receptor form, soluble TREM2 (sTREM2) also participates in signal regulation (**Figure 1C**). The production of sTREM2 primarily depends on cleavage by metalloproteases (59, 64) or alternative splicing mechanisms (65). Specifically, the extracellular domain of TREM2 is cleaved by ADAM10 and ADAM17—members of the ADAM protease family—releasing sTREM2. Notably, the primary cleavage site of ADAM17 is located between histidine 157 (His 157) and serine 158 (Ser 158) (64). The short cytoplasmic domain of TREM2 is cleaved by γ-secretase to release the TREM2 intracellular domain (ICD) (59); however, the functions of TREM2 ICD remain unclear. In summary, the activation and regulation of the TREM2 pathway rely on interactions between multiple molecules. Therefore, it is necessary to conduct in-depth research on its role in macrophages and its relationship with immune-related diseases.

## 4 The Functions of TREM2

### 4.1 Phagocytosis

**Figure 2:**
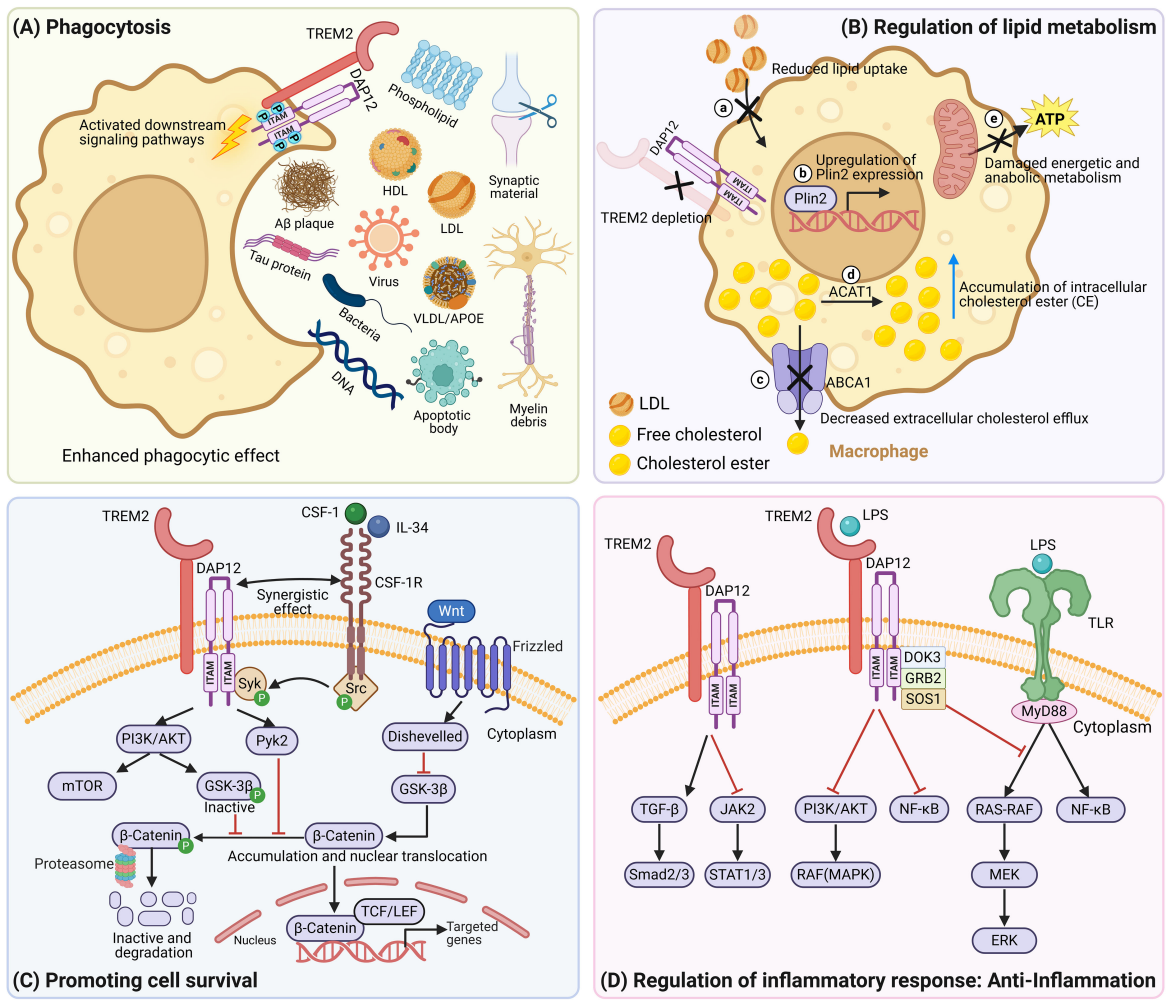
The functions of TREM2. **(A)** The extracellular domain of TREM2 contains a V-type immunoglobulin domain, that allows TREM2 to interact with multiple ligands, including membrane phospholipids, lipoprotein (LDL, HDL, VLDL), lapidated apolipoproteins APOE, Aβ plaque, tau protein, myelin debris, apoptotic body, synaptic materials, bacteria, viruses, and DNA. Upon binding to these ligands, TREM2 activates downstream signaling pathways, ultimately leading to the activation of proteins associated with phagocytosis and the reorganization of the cytoskeleton, thereby enhancing the phagocytic capacity of macrophages for these ligands. This process plays a crucial role in the clearance of pathogens, apoptotic cells, abnormal protein deposits, and the maintenance of tissue homeostasis by macrophages. **(B)** TREM2-deficient macrophages exhibit reduced lipid uptake, upregulation of Plin2 expression, and downregulation of ABCA1 expression, leading to increased formation of lipid droplets and reduced cholesterol efflux. The increased intracellular cholesterol is subsequently esterified into cholesterol ester by ACAT1. Additionally, TREM2 deficiency impairs energy and anabolic metabolism in macrophages. **(C)** The TREM2/DAP12 signaling pathway intersects with the CSF1R signaling pathway to promote macrophages survival and proliferation by activating the PI3K/AKT-mTOR and Wnt/β-catenin signaling pathways. **(D)** After binding to LPS, TREM2 negatively regulates the TLR signaling pathway and downregulates the PI3K/AKT and NF-κB signaling pathways to alleviate inflammatory responses. Furthermore, TREM2 effectively reduces neuroinflammation in Alzheimer’s disease mouse models by inhibiting the JAK2-STAT1/STAT3 signaling pathway. Please note that, upon activation, TREM2 promotes inflammatory responses through downstream NF-κB and MAPK signaling pathways (**Figure 1**). However, depending on cell type or specific stimulation conditions, TREM2 can also exhibit anti-inflammatory effects. **(D)** highlights the mechanisms underlying its anti-inflammatory role. Image created with BioRender.com.

TREM2 initiates the phagocytic process by recognizing and binding to target ligands, including anionic lipids (66), membrane phospholipids (67), lipoprotein (68), lapidated apolipoproteins (66, 69), synaptic materials (4, 15), amyloid-β (Aβ) (16–19, 61, 66, 70, 71), tau protein (72), myelin debris (73), as well as bacterial and virus products (74) and DNA (59) that emerge under tissue damage (**Figure 2A**). A seminal study utilized transfection technology to introduce the TREM2-DAP12 complex into Chinese hamster ovary (CHO) cells, providing the first demonstration of the phagocytic capability of the TREM2-DAP12 complex against bacteria (74). Other studies have indicated that TREM2^+^ macrophages play a critical phagocytic role across various disease models. In Alzheimer’s disease (AD), TREM2 participates in the clearance of amyloid plaques via phagocytosis (61). In glioblastoma (GBM) (75) and lung cancer (76), TREM2 also regulates the tumor microenvironment (TME) by clearing apoptotic tumor cell debris through phagocytic mechanisms. Additionally, TREM2 is involved in the clearance of necrotic myocardial cells after acute myocardial infarction (55) and the removal of lipid-rich apoptotic hepatocytes in non-alcoholic steatohepatitis (NASH) (25). Furthermore, TREM2 is essential for microglia-mediated synaptic pruning during the early stages of brain development, and TREM2 deficiency potentially leads to reduced microglial activation, that affects normal elimination mechanisms of synapses. Prior studies have shown that *Trem2^-/-^* adult mice exhibit defects in social behavior and alterations in brain connectivity. Additionally, in patients with autism, there is a negative correlation between TREM2 protein levels and symptom severity, suggesting that TREM2 may play a protective role in neurodevelopmental disorders, such as autism (4, 15). Importantly, phagocytosis mediated by TREM2^+^ macrophages requires the presence of a full-length TREM2 receptor, and ADAM protease inhibitors maintain the phagocytic function of TREM2 by inhibiting its shedding (64). However, the TREM2 H157Y variant enhances TREM2 cleavage, resulting in reduced cell surface levels of TREM2 and consequently impairing TREM2-dependent phagocytosis (64). Other TREM2 variants, such as the TREM2 R47H variant, reduce the affinity of TREM2 for ligands such as Aβ (77), APOE (66), CLU/APOJ (66), and certain lipids (67) due to the substitution of arginine with histamine at position 47. Similarly, TREM2 R62H variant substitutes arginine with histidine at position 62, disrupting TREM2’s recognition of APOE without affecting its binding to lipid ligands (66). Furthermore, the TREM2 p.T66M mutation causes structural distortion or improper folding of the TREM2 protein, thereby reducing the phagocytic activity of bone marrow-derived macrophages (BMDMs) in mice towards Aβ. Conversely, the TREM2 T96K variant shows increased binding to ligands and potentially exerts a protective effect against AD (58). In summary, TREM2 and its variants play complex roles in regulating the phagocytic function of macrophages, and investigations into their mechanisms of action have provided new insights into the pathogenesis of diseases associated with TREM2.

### 4.2 Regulation of lipid metabolism

Extensive research has established TREM2 as the primary modulator of lipid metabolism (**Figure 2B**). Researchers fed mice a diet containing 0.2% cuprizone (CPE) to mimic conditions of chronic demyelination and found that TREM2-deficient microglia exhibited reduced clearance of myelin cholesterol and decreased lipid hydrolysis, that led to the abnormal accumulation of cholesterol ester (CE). Strategies using acetyl-CoA acetyltransferase 1 (ACAT1) inhibitors and LXR agonists can rescue CE accumulation (78). Furthermore, in the context of ischemic stroke, the expression of perilipin-2 (Plin2) is upregulated, and the expression of ATP-binding cassette transporter A1 (ABCA1) is downregulated, leading to increased lipid droplet formation and impaired cholesterol clearance in TREM2-deficient microglia (79). In atherosclerosis, cholesterol accumulation in foam macrophages is a key factor in plaque formation and disease progression. The direct impact of TREM2 deficiency may involve a reduction in the expression of cholesterol transporters, such as ABCA1 and ABCG1, that impairs cholesterol efflux, compromises cell survival, which seems to exacerbate the pathological process of atherosclerosis (80). Interestingly, TREM2 deficiency is also associated with decreased lipid uptake capacity in macrophages, suggesting a reduction in the total amount of cholesterol and other lipids entering the macrophages (80, 81). Consequently, although cholesterol outflow is impeded, the overall intracellular cholesterol levels may remain relatively low. This indirectly aids in maintaining cholesterol homeostasis, reduces the lipid load of plaques, and ultimately slows the progression of atherosclerosis. In addition to its effect on lipid metabolism, TREM2 also affects energy and anabolic processes. Prior studies have revealed that in Alzheimer’s disease, TREM2-deficient microglia present impaired activation of the mTOR signaling pathway, resulting in decreased ATP levels and obstructed biosynthetic pathways that in turn, trigger an abnormal increase in microglial autophagy. Dietary supplementation with cyclocreatine can reduce the abnormal autophagy of microglia and improve their clearance of Aβ plaques (82). Additionally, the TREM2 T66M variant can impair microglial function, leading to reduced cerebral blood flow and glucose metabolism (70). The above examples further emphasize the crucial role of TREM2 in maintaining metabolic homeostasis.

### 4.3 Promoting cell survival

Extensive researches have highlighted the role of the TREM2-DAP12 complex in promoting macrophage survival and proliferation through intricate signaling cascades (**Figure 2C**). For example, colony-stimulating factor 1 receptor (CSF1R) can crosstalk with TREM2-DAP12 signaling through ITAM phosphorylation by Src kinase to activate PI3K/AKT signaling, that in turn activates mTOR signaling and imparts vital survival signals to macrophages (5, 7, 59, 80). TREM2 deficiency leads to increased macrophage apoptosis and decreased survival by inhibiting the Akt/mTOR signaling pathway. Cyclocreatine supplementation significantly improves the survival rates of TREM2-deficient macrophages (83). Moreover, activated AKT can also phosphorylate glycogen synthase kinase-3β (GSK-3β), resulting in GSK-3β inactivation, thereby reducing the phosphorylation and subsequent ubiquitination and proteasome-mediated degradation of β-catenin. This ultimately leads to the accumulation and stabilization of β-catenin and the further translocation of β-catenin into the nucleus (42, 67, 84). Notably, this process also involves the tyrosine kinase Pyk2, that inhibits β-catenin phosphorylation (5). In addition, activated Wnt signaling leads to the recruitment of disheveled (Dvl), that inhibits GSK3-β and causes the subsequent nuclear translocation of β-catenin (84, 85). The above-mentioned m-TOR and Wnt-β-catenin signaling pathways effectively promote microglial survival and proliferation. Furthermore, in AD mouse models and patients with AD carrying TREM2 risk variants, TREM2-deficient microglia exhibit abnormal autophagic activity, suggesting that TREM2 plays a key role in regulating microglial autophagy homeostasis (59, 82). In summary, TREM2 plays an indispensable role in microglial survival through a complex signaling network. The dysfunction of TREM2 affects immune homeostasis and is closely linked to the pathogenesis of neurodegenerative diseases.

### 4.4 Regulation of inflammatory response

#### 4.4.1 Anti-inflammation

Numerous studies have revealed that TREM2 acts as a negative regulator of inflammatory responses (**Figure 2D**). In experiments using mouse models of simulated infection, researchers have found that TREM2 can negatively regulate the TLR4 signaling pathway to inhibit the inflammatory response in early stage gram-negative bacteremia (86). Furthermore, mechanistic studies have shown that following LPS stimulation, the anti-inflammatory adaptor protein DOK3 is phosphorylated, associates with DAP12, and interacts with Grb2 and SOS1 to inhibit the TLR4-mediated activation of the MAPK signaling pathway at the RAF level, leading to a reduction in inflammatory actions (5, 7). Moreover, TREM2 can mitigate the LPS-induced neuroinflammatory response in microglia by downregulating the PI3K/AKT and NF-κB signaling pathway (87). In AD mouse models, TREM2 overexpression effectively reduces neuroinflammation and improves cognitive function in mice by inhibiting the JAK2-STAT1/STAT3 signaling pathway (88). In ischemic brain injury, TREM2 promotes the polarization of M2 anti-inflammatory microglia by upregulating the TGF-β/Smad2/3 signaling pathway, thereby alleviating neural damage after cerebral ischemia (79). During the repair process following acute colonic mucosal injury, TREM2 signaling promotes the activation of M2 type macrophages by modulating the balance between Th1 and Th2 cytokines, thereby enhancing the anti-inflammatory response and ultimately facilitating epithelial cell proliferation and wound healing (89). In a high-fat diet (HFD)-induced diabetic mouse model, dendritic complexity and spine density in the CA1 region of the hippocampus were reduced. Overexpression of TREM2 in the hippocampus, mediated by adeno-associated virus (AAV)-mediated gene delivery, partially restored these structures and significantly improved diabetes-associated cognitive dysfunction without affecting body weight or glucose homeostasis in the mice. Specifically, TREM2 overexpression markedly suppressed the activation of the NF-κB signaling pathway in the hippocampal region and reduced the expression of pro-inflammatory cytokines (such as IL-1β, TNF-α, and TLR-4), thereby inhibiting neuroinflammation (90). Another study demonstrated that TREM-2 suppresses inflammatory responses by negatively regulating the p38 MAPK signaling pathway, alleviating neuroinflammation and cognitive impairment caused by the combined effects of diabetes mellitus (DM) and chronic cerebral hypoperfusion (CCH) on the organism (91).

#### 4.4.2 Pro-inflammation

However, in contrast to its predominantly anti-inflammatory nature, TREM2 exhibits pro-inflammatory effects under certain conditions. In a study on neuropathic pain, the TREM2-DAP12 complex can induce the expression of TNF-α, IL-6, and pain-related genes in the early stages after injury, thereby intensifying neuropathic pain (92). In acquired cholesteatoma, there is an increased expression of inflammatory cytokines, including IL-1β, TNF-α, and IL-6, as well as matrix metalloproteinases MMP-2, MMP-8, and MMP-9, that is positively associated with the elevated levels of TREM2 (44). In the middle cerebral artery occlusion (MCAO) mouse model, mRNA expression of TREM2 in ischemic brain tissue is significantly upregulated at both 7 days and 28 days post-injury. The expression of pro-inflammatory cytokines (TNF-α, IL-1α, IL-1β) and chemokines (CCL2, CCL3), as well as the chemokine receptor CX3CR1, is reduced at 7 days after stroke in TREM2-KO mice, along with attenuated activation of microglia. Furthermore, the infiltration of CD3^+^ T cells is decreased at 28 days after stroke in TREM2-KO mice. All of these factors contribute to an overall attenuated inflammatory response (93). During Plasmodium liver infection in mice, the expression of TREM2 in Kupffer cells is closely associated with resistance to the parasite. In *Trem2^−/−^* mice, Kupffer cells exhibit an anti-inflammatory activation profile following Plasmodium infection, characterized by high expression of Arg1 mRNA and low expression of pro-inflammatory cytokines such as IL-6, IL-1β, TNF-α, and CD68 mRNA, which results in a higher liver parasite burden (27). In a rhesus macaque model infected with SARS-CoV-2, the proportion of alveolar macrophages decreased, while the proportions of CD163^+^ MRC1^-^ and TREM2^+^ macrophages increased. These infiltrating macrophages drive the initiation and exacerbation of lower respiratory tract inflammation by producing inflammatory cytokines such as IL-6, TNF, and IL-10 (94). As previously discussed, TREM2 contributes to the inflammatory response in IBD patients by augmenting the functional activity of dendritic cells (34). Collectively, these findings suggest that the role of TREM2 in inflammatory responses may be associated with the nature of its ligand, disease stage, and immune environment.

## 5 The Effect of TREM2+ macrophages on disease development

**Figure 3:**
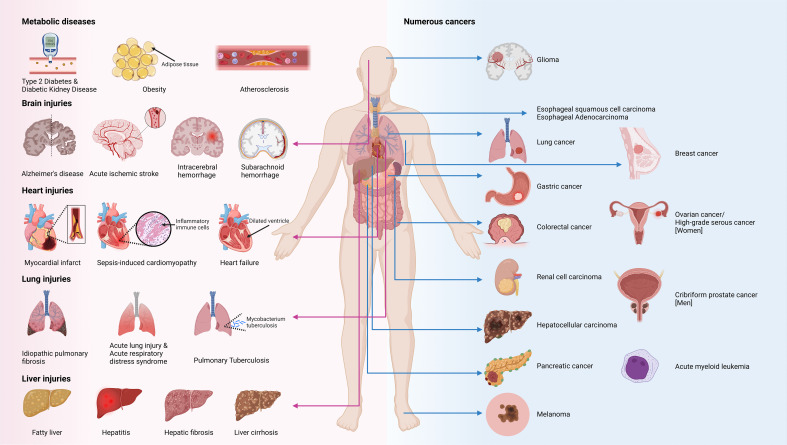
TREM2 as a marker in immune related diseases. Metabolic syndrome refers to a complex cluster of metabolic disorders that encompasses various diseases, such as obesity, diabetes, atherosclerosis, and fatty liver, that elevate the risk of multiple organ damage, including brain, heart, lung, and liver injuries. TREM2, a receptor that is primarily known for its anti-inflammatory properties, may play a protective role in multiple noncancerous diseases. However, in various cancers, TREM2 promotes tumorigenesis and cancer progression. Notably, TREM2 may exert dual functions in liver injury, hepatocellular carcinoma, glioma, ovarian cancer and melanoma, whereas in acute myeloid leukemia, TREM2 can halt disease progression. Image created with BioRender.com.

As mentioned earlier, TREM2 is a receptor that is primarily expressed on various macrophages and is crucial for maintaining the functional homeostasis and anti-inflammatory effects of these cells. In non-cancerous conditions, TREM2 is recognized for its protective role. However, TREM2 paradoxically switches to a tumorigenic role in most cancers, where it contributes to cancer progression and aggression. Notably, in certain cancers, TREM2 exhibits duality depending on the type and stage of the cancer and may either promote or inhibit tumor growth. Therefore, we next investigated the role of TREM2^+^ macrophages in the development of non-cancerous and cancerous diseases, including metabolic, neurological, pulmonary, and cardiovascular diseases and cancers (**Figure 3**, **Table 2**).

**Table 2:** The effect of TREM2^+^ macrophages on disease development.

Diseases	Functions	Signaling pathways	References
Type 2 Diabetes	Protective	Inhibiting the NF-κB signaling pathway	(90)
	Protective	Negatively regulating the p38 MAPK signaling pathway	(91)
Renal injury	Protective	Inhibiting the JAK-STAT signaling pathway	(83)
Obesity	Protective	N/A	(97–99)
NASH/NAFLD	Dual roles/Protective	N/A	(100)
NASH	Dual roles/Pathogenic	LD-MS4A7-NLRP3 triggers TREM2^+^ macrophage induction	(101)
Cholestatic liver injury	Protective	N/A	(31)
Acute and chronic liver injury	Protective	Inhibition of TLR4 mediated inflammation	(28, 29)
Atherosclerosis	Dual roles/Protective	N/A	(104)
	Dual roles/Protective	Activating the PI3K/AKT/mTOR signaling pathway	(105)
	Dual roles/Pathogenic	Upregulating of CD36	(81)
Alzheimer’s disease	Protective	Activating the PI3K/AKT/mTOR; Vav and PLCγ2 signaling pathways	(16–19, 61, 66, 70, 71)
Acute ischemic stroke	Protective	TREM2‐IGF1 axis	(107)
	Protective	Activating the TGF-β1/Smad2/3 signaling pathway	(79)
Intracerebral hemorrhage	Protective	Activating the PI3K/AKT signaling pathway	(108)
	Protective	Inhibiting the NF-κB and MAPK signaling pathways	(109)
Subarachnoid hemorrhage	Protective	Elevating IRAK3 expression	(110)
Idiopathic pulmonary fibrosis	Pathogenic	Activating the STAT6	(114)
ALI/ARDS	Protective	Rhein activates NFATc1 and upregulates TREM2 expression while promoting M2 macrophage polarization	(115)
	Protective	GSP upregulates TREM2 expression and activates the PI3K/Akt signaling pathway while promoting M2 macrophage polarization	(116)
Tuberculosis	Unclear	N/A	(117)
Myocardial infarction	Protective	Activating the SYK-SMAD4	(55)
SICM	Protective	N/A	(121, 122)
Heart failure	Protective	N/A	(123)
Hepatocellular carcinoma	Dual roles/Enhance tumorigenesis	N/A	(22)
	Dual roles/Suppress tumorigenesis	Targeting the PI3K/Akt/β-catenin	(102)
	Dual roles/Suppress tumorigenesis	N/A	(30)
Glioma	Dual roles/Enhance tumorigenesis	N/A	(111)
	Dual roles/Enhance tumorigenesis	Activating the TLR4/Akt signaling pathway	(112)
	Dual roles/Suppress tumorigenesis	N/A	(75, 113)
Lung cancer	Enhance tumorigenesis	Activating the Syk signaling	(35)
	Enhance tumorigenesis	N/A	(76, 118, 119)
ESCC	Enhance tumorigenesis	N/A	(124)
EAC	Enhance tumorigenesis	Activating the TYROBP-Syk and JAK-STAT3	(125)
Gastric cancer	Enhance tumorigenesis	Activating the PI3K/AKT	(127)
Colorectal cancer	Enhance tumorigenesis	N/A	(47)
Pancreatic cancer	Enhance tumorigenesis	N/A	(129)
Renal cell carcinoma	Enhance tumorigenesis	Regulating the PI3K/Akt	(130)
Cribriform prostatic cancer	Enhance tumorigenesis	N/A	(133)
Ovarian cancer	Dual roles/Enhance tumorigenesis	N/A	(134)
Ovarian cancer/HGSC	Dual roles/Suppress tumorigenesis	N/A	(135)
Breast cancer	Enhance tumorigenesis	N/A	(47, 136–139)
Melanoma	Dual roles/Enhance tumorigenesis	N/A	(140)
	Dual roles/Suppress tumorigenesis	Activating the caspase 3/GSDME	(141)
Melanoma/SKCM	Dual roles/Suppress tumorigenesis	N/A	(142)
Sarcoma	Enhance tumorigenesis	N/A	(47)
Acute myeloid leukemia	Suppress tumorigenesis	IL-34-TREM2 binding phosphorylates Rasal3, thereby inhibiting ERK1/2	(143)

NASH, Nonalcoholic Steatohepatitis.

NAFLD, Nonalcoholic Fatty Liver Disease.

ALI, Acute lung injury.

ARDS, Acute respiratory distress syndrome.

SICM, Sepsis-induced cardiomyopathy.

ESCC, Esophageal squamous cell carcinoma.

EAC, Esophageal Adenocarcinoma.

HGSC, High-grade serous cancer.

SKCM, skin cutaneous melanoma.

### 5.1 Metabolic diseases

It is well known that metabolic abnormalities in the body, such as hypertension, hyperglycemia, and hyperlipidemia—commonly referred to as the “three highs”—are closely associated with the risk of developing various diseases. Among the numerous metabolism-related conditions, type 2 diabetes (T2D) not only represents a quintessential metabolic disorder but also significantly increases the risk of cognitive impairment in patients (57, 90, 91). In the context of type 2 diabetes-related cognitive impairment (TDACD), TREM2 plays a pivotal role in modulating neuroinflammation and neuronal damage by regulating macrophage phagocytosis and neuronal survival (57). As previously mentioned, the overexpression of TREM2 attenuated the phosphorylation of NF-κB p65 and IkB-α in the hippocampus of HFD-fed mice and reduced the mRNA levels of TLR-4 and IL-1β, indicating that TREM2 exerts its neuroprotective effects by inhibiting the NF-κB pathway and neuroinflammation (90). Furthermore, TREM2 plays an anti-inflammatory role under diabetic and CCH conditions by suppressing the p38 MAPK signaling pathway (91). These mechanisms provide new insights into understanding the protective role of TREM2 in cognitive dysfunction induced by diabetes. Diabetic kidney disease (DKD) is one of the most common complications of diabetes and a leading cause of chronic renal failure. Researchers have found that *Trem2^-/-^* mice exhibited more severe kidney damage under high-fat diet induction, indicating a protective role of TREM2^hi^ macrophages in DKD (95). In the UUO mouse model, TREM2 deficiency promotes macrophage polarization towards both M1 and M2 phenotypes via the activation of the JAK-STAT pathway, leading to more severe tubular injury, higher levels of inflammatory cytokines (TNF-α, IL-1β, IL-6), and more extensive interstitial fibrosis (83). TREM2 is also closely associated with insulin resistance and type 2 diabetes (T2D) (96, 97). For example, scientists have found that *Trem2^-/-^* mice exhibit insulin resistance, glucose intolerance, adipocyte hypertrophy, and fat accumulation in diet-induced obesity models. More importantly, these mice failed to form typical crown-like structures (CLS), that are crucial for clearing apoptotic adipocytes and lipid debris during obesity (97–99).

Furthermore, the role of TREM2 in obesity-induced fatty liver disease should not be overlooked. Prior studies have shown that *Trem2^-/-^* mice exhibit more severe hepatic steatosis after long-term high-fat diet feeding (99). In the intricate progression of liver diseases, hepatitis, liver cirrhosis and liver cancer constitute a well-known “trilogy”. Epidemiological investigations have revealed that approximately 20% of patients with non-alcoholic fatty liver disease (NAFLD) progress to non-alcoholic steatohepatitis (NASH), a process accompanied by chronic liver inflammation, fibrosis, and ultimately, potential evolution into cirrhosis and liver cancer. Current research highlights the crucial involvement of macrophages in the evolution of NAFLD/NASH (25, 26, 96, 100). For example, in chemical cholestasis mouse model, TREM2 demonstrates a positive effect in reducing liver inflammation and protecting hepatocytes from damage (31). Conversely, the absence of TREM2 triggered mitochondrial dysfunction and impaired energy supply in hepatic macrophages while resulting in severe complications, such as sepsis (100). In both the acetaminophen (APAP)-induced acute liver injury mouse model and the CCl4-induced chronic liver injury mouse model, *Trem2^-/-^* mice exhibited enhanced production of pro-inflammatory cytokines (IL-6 and TNF-α) and chemokines (MCP-1) from Kupffer cells and hepatic stellate cells following TLR4 stimulation, thereby exacerbating hepatic damage. In contrast, overexpression of TREM2 demonstrated a protective effect by attenuating this liver injury (28, 29). Additionaly, the function of TREM2 closely relates to nutritional status. Long-term malnutrition has been shown to impair TREM2-dependent efferocytosis, thereby accelerating NASH progression (26). However, TREM2 overexpression is not always beneficial. In certain situations, such as when the lipid droplet (LD)-MS4A7-NLRP3 inflammasome axis is activated, TREM2 can promote the induction of TREM2^+^ macrophages, thereby accelerating hepatic inflammatory responses and fibrosis, and driving the deterioration of NASH (101). Scientists have identified a population of cells within hepatocellular carcinoma (HCC) tumor tissues that exhibit characteristics similar to lipid-associated macrophages (LAMs), that have been termed TREM2^+^ LAM-like cells. These cells play an immunosuppressive role in the HCC tumor microenvironment and are closely associated with poor patient prognosis (22). However, in a mouse model of HCC induced by diethylnitrosamine (DEN), the absence of TREM2 led to a higher incidence of HCC and more severe liver damage, inflammation, and oxidative stress (30). Furthermore, in a mouse model of HCC pulmonary metastasis, TREM2 was found to suppress HCC metastasis and tumorigenesis through targeting the PI3K/Akt/β-catenin signaling pathway (102). In summary, TREM2 plays a distinct dual role in liver injury and HCC; however, its specific mechanisms require further investigation to be fully understood.

Recently, TREM2 has garnered significant attention for its role in atherosclerosis (AS). Using advanced scRNA-seq trajectory analysis and whole-genome CRISPR screening techniques, researchers have successfully identified a novel cell population of TREM2^hi^ macrophages (103). The latest research findings indicate that TREM2 not only promotes the proliferation and survival of foam macrophages but also reduces the formation of necrotic cores within plaques, thereby maintaining the stability of atherosclerotic plaques (104). Furthermore, the TREM2 agonist antibody AL002a promotes the uptake of oxidized low-density lipoprotein (oxLDL) by foam macrophages, enhances cell survival, and facilitates cholesterol efflux by activating Syk kinase and the downstream PI3K/AKT/mTOR pathway (105). However, as atherosclerosis progresses, excessive accumulation and death of foam macrophages regulated by TREM2 may invoke new risk factors for plaque instability and rupture. Related studies have also indicated that TREM2 accelerates the transformation of vascular smooth muscle cells and macrophages into foam cells by upregulating the expression of the scavenger receptor CD36, thus enlarging plaque volume and exacerbating the progression of atherosclerosis (81).

### 5.2 Neurological diseases

Alzheimer’s disease (AD) is one of the most common neurodegenerative diseases worldwide. Recently, TREM2, a key lipid sensor in microglia, has emerged as a crucial player in AD and other neurological diseases (67). TREM2 not only maintains the number and function of microglia in the aging brain but also responds actively to demyelinating injuries, thus serving as an important guardian of nervous system homeostasis. In the pathological context of AD, TREM2 binds to lipids or Aβ on the surface of apoptotic cells through its extracellular domain and effectively clears harmful substances (16–19, 61, 66, 70, 71). Notably, TREM2 also plays distinct roles at different stages of AD. In the early and middle stages, increasing TREM2 expression can reprogram the reactivity of microglia, reduce Aβ burden, and improve neuropathology and behavioral deficits. However, in the late stage, microglia exacerbate neurodegeneration by phagocytosing the tau protein and accelerating its phosphorylation (72). These findings reveal the complexity and duality of microglia and neuroinflammation in AD progression. In addition to TREM2, CD33 is considered a risk factor for AD. As an inhibitory receptor that acts upstream of TREM2, CD33 influences AD susceptibility by regulating the phagocytic activity of microglia (63). The 5xFAD mouse model, which carries five mutations associated with familial Alzheimer’s disease (FAD), closely mimics human AD pathology. Related research has also shown that the deletion of the CD33 gene can alleviate Aβ plaque burden and improve cognitive function in 5xFAD mice. However, these effects were abrogated when TREM2 was additionally deleted, further highlighting the central role of TREM2 in the pathogenesis of AD. Inhibition of CD33 and/or enhancement of TREM2 activity represent potential therapeutic strategies for AD (106).

In addition to AD, TREM2 exerts significant neuroprotective effects in various neurological diseases. Particularly in acute ischemic stroke (AIS), researches have shown that the TREM2-IGF1 axis (107) and the TREM2-TGF-β1/Smad2/3 axis (79) exert neuroprotective effects after ischemic stroke by regulating the functions and metabolic characteristics of microglia. The neuroprotective effects of TREM2 are not limited to AIS but also show significant benefits in intracerebral hemorrhage (ICH) and subarachnoid hemorrhage (SAH). Within 24 h after ICH, the expression of endogenous TREM2 significantly increased and reached its peak. Furthermore, On one hand, TREM2 improves neurological function by activating the PI3K/AKT signaling pathway (108). On the other hand, TREM2 inhibits the activation of TLR4, subsequently blocking the activation of NF-κB and MAPK signaling pathways, that play crucial roles in neuroinflammation and neuronal apoptosis after ICH (109). Additionally, at 48 h post-SAH, TREM2 expression reaches its peak. TREM2 activates the phagocytic function of microglia and inhibits the inflammatory response, thereby improving neuroinflammation and prognosis in patients with SAH (110).

TREM2 plays a protective role in various neurological diseases mentioned above; however, it exhibits a dual role in glioma. GBM, the most common primary brain tumor in adults, poses a significant challenge in the treatment of the CNS diseases because of its high aggressiveness and low sensitivity to radiotherapy, chemotherapy, and immunotherapy. By analyzing RNA sequencing data from the TCGA (The Cancer Genome Atlas) and CGGA (Chinese Glioma Genome Atlas) databases, Yu M et al. have revealed that TREM2 expression is significantly higher in mesenchymal gliomas compared to other subtypes, and its high expression is closely associated with poor prognosis in glioma patients. Mesenchymal glioma, a distinct subtype of glioma, is characterized by mesenchymal transition, exhibiting stronger invasiveness and migratory capabilities (111). Additionally, Qiu H et al. established a TREM2 stably knocked-down glioblastoma (GBM) cell line (GBM TREM2−KD) using short hairpin RNA (shRNA) technology. *In vitro* experiments confirmed a positive feedback relationship between TREM2 and high-mobility group box 1 (HMGB1), where TREM2 maintains high levels of HMGB1 expression, and HMGB1, by binding to TLR4, further activates the downstream PI3K/Akt signaling pathway. This mechanism enhances the radiotherapy resistance of GBM and promotes its immune escape capability (112). These findings suggest that TREM2 plays a role in promoting tumor progression in GBM. However, other studies have shown that TREM2 exhibits distinct immune regulatory roles in brain gliomas and peripheral tumors, and it primarily exerts a protective function in brain gliomas. Peshoff et al. found that in an intracranial glioma model, stereotactic injection of luciferase-labeled GL261 cells (GL261-luc) into the brains of mice revealed that *Trem2^−/−^* mice, although showing no significant difference in survival rates, exhibited higher tumor burden and faster tumor growth, suggesting that TREM2 may inhibit the growth of intracranial gliomas. In contrast, in a peripheral tumor model, subcutaneous injection of luciferase-labeled CT-2A cells (CT-2A-luc) resulted in significantly smaller tumor volumes in *Trem2^−/−^* mice, indicating that TREM2 may promote the growth of peripheral tumors (75). Furthermore, Zhong et al. found that in a CNS tumor model, intracranial injection of the EO771 breast cancer cell line into mice resulted in accelerated tumor growth and reduced survival rates in *Trem2^−/−^* mice, further confirming the antitumor role of TREM2 in CNS tumors. However, in a peripheral tumor model, subcutaneous injection of GL261 or EO771 cells led to slower tumor growth in *Trem2^−/−^* mice, accompanied by a shift in the tumor microenvironment toward a pro-inflammatory state, again suggesting that TREM2 promotes tumor growth in peripheral tumors (113). These results indicate that the antitumor effects of TREM2 in CNS gliomas occur specifically within the unique environment of the CNS. The underlying mechanism may involve CNS-enriched lipid signals, such as sphingomyelin, binding to TREM2 and activating the antitumor response of microglia (113). This appears to contradict the previously described role of TREM2 in promoting tumor progression in GBM, and the specific mechanisms underlying this discrepancy require further investigation.

### 5.3 Pulmonary diseases

In recent years, TREM2 has garnered widespread attention owing to its role in the pathogenesis of various pulmonary diseases, including chronic obstructive pulmonary disease (COPD) (24), idiopathic pulmonary fibrosis (IPF) (114), acute lung injury (ALI) (115, 116), acute respiratory distress syndrome (ARDS) (115, 116), and tuberculosis (TB) (117). In habitual smokers, the transcriptional level of TREM2 in alveolar macrophages increases significantly. The TREM2-DAP12 complex can regulate the chemotaxis and recruitment of alveolar macrophages to the lungs mediated by CCL2, thus participating in the repair process of damaged lung tissue of COPD. However, its specific mechanism remains incompletely understood (24). IPF is a chronic, progressive, and fatal interstitial lung disease; however, its pathogenesis remains unclear. Recent research has shown that in patients with IPF and bleomycin (BLM)-induced pulmonary fibrosis in mouse models, the TREM2 expression is significantly elevated and associated with poor prognosis. Further research has revealed that TREM2 deficiency can inhibit STAT6 activity and reduce anti-inflammatory macrophage polarization, thereby exerting a protective effect against pulmonary fibrosis (114). ALI is characterized by widespread pulmonary inflammation and tissue damage, which may progress to ARDS. Rhein, a component of traditional Chinese medicine, activates NFATc1 and interacts with it, enabling NFATc1 to bind to the TREM2 promoter region and promote TREM2 expression. The upregulated TREM2 inhibits NF-κB activation and nuclear translocation, thereby suppressing M1 macrophage polarization and alleviating lung inflammation. In survival experiments, mice treated with 100 mg/kg of Rhein showed significantly higher long-term survival rates, indicating that Rhein provides long-term protective effects for mice with ALI/ARDS (115). Additionally, in a mouse model of LPS-induced ALI via intratracheal instillation, pretreatment with different doses of grape seed proanthocyanidin (GSP) significantly alleviated LPS-induced pathological lung tissue damage, including alveolar congestion, interstitial edema, alveolar wall thickening, and inflammatory cell infiltration. Specifically, GSP upregulated TREM2 expression, thereby activating the downstream PI3K/Akt signaling pathway and promoting the transition of macrophages to an anti-inflammatory phenotype (116). TB is a global infectious disease caused by *Mycobacterium tuberculosis (Mtb)*. TREM2 and CD163 are highly expressed in tuberculous granulomas and may be associated with the formation of tuberculosis lesions and latent infections (117).

Despite its protective role against various pulmonary inflammatory diseases, TREM2 primarily promotes lung cancer progression. TREM-2 promotes the secretion of IL-10 by activating the Syk pathway, thereby enhancing immune suppression and inhibiting the proliferation and function of T cells (35). Clinical investigations have revealed that the expression level of TREM2 on pulmonary macrophages is increased in lung cancer patients and is closely positively correlated with the pathological stage and lymph node metastasis of lung cancer (35). Furthermore, patients with low TREM2^+^ TAMs infiltration exhibit a significantly higher response rate (31.58%) to PD-1-based immune checkpoint blockade (ICB) therapy, compared to those with high TREM2^+^ TAMs infiltration (14.29%) (118). In non-small cell lung cancer (NSCLC), TREM2^+^ TAMs are specifically recruited to the tumor tissue of lung cancer through the CCL2-CCR2 axis (119). They not only produce anti-inflammatory cytokines but also contribute to the immune evasion of NSCLC by diminishing the anti-tumor activity of CD8^+^ T cells and promoting the differentiation of FOXP3^+^ T regulatory cells (118). Additionally, TREM2 can effectively suppress the activity of NK cells by finely regulating the IL-18/IL-15 signaling pathway. It is noteworthy that when TREM2 was knocked out or interfered with anti-TREM2 antibody, the recruitment and activation of NK cells were significantly promoted, thereby effectively inhibiting the occurrence and development of lung cancer (76).

### 5.4 Cardiovascular diseases

In cardiovascular disease research, especially in the fields of myocardial infarction (MI) (55, 120), sepsis-induced cardiomyopathy (SICM) (121, 122), and heart failure (HF) (123), the role of TREM2 in cardiac macrophages is gaining increasing attention. In MI, TREM2 expression levels exhibit a pronounced time-dependent upward trend in cardiac macrophages and peak in the late stages of MI (120). Recent research has shown that TREM2 enhances the efferocytosis of macrophages and triggers the TREM2-SYK-SMAD4 signaling cascade, thereby inhibiting the transcriptional activity of SLC25A53 and affecting NAD^+^ transport and the tricarboxylic acid (TCA) cycle in the mitochondria. This ultimately promotes the generation of itaconate, that is beneficial for the recovery of cardiac function and structure after MI (55). Furthermore, cardiac-resident macrophages characterized by high TREM2 expression (TREM2^hi^ Mac1) promote the restoration of cardiac function after SICM by maintaining the homeostasis of cardiomyocyte mitochondria (121, 122). It is worth noting that scientists have also confirmed the protective role of TREM2 in a deoxycorticosterone acetate (DOCA)-salt-induced mouse model of hypertensive heart failure using single-cell sequencing technology. *TREM2^-/-^* mice exhibit obvious symptoms, such as cardiac hypertrophy, diastolic dysfunction, renal injury and decreased cardiac capillaries density (123), further highlighting the significant role of TREM2 in cardiovascular diseases.

### 5.5 Numerous cancers

TREM2 expression is closely related to immune cell infiltration in the tumor microenvironment, particularly in macrophages. An increasing number of studies have indicated that TREM2 upregulation is a significant marker of immune suppression in the tumor microenvironment and promotes tumor progression in a vast majority of cancers (47).

Notably, a prior scRNA-seq analysis indicated that TREM2^+^ TAMs were significantly enriched in esophageal squamous cell carcinoma (ESCC) tumor tissues and were closely associated with shorter OS in patients with ESCC (124). Previous study has shown that TREM2 maintains the growth of esophageal adenocarcinoma (EAC) cells by activating the TYROBP-Syk and JAK-STAT3 signaling pathways. Targeting TREM2-related signaling pathways with fostamatinib R788 can induce EAC cell death and growth arrest, reduce tumor burden, and demonstrate anti-tumor effects in xenografted mouse models (125). Furthermore, recent studies have revealed that the expression of TREM2 in gastric cancer tissues is significantly higher than that in adjacent normal tissues (126–128) and that elevated TREM2 expression closely associates with multiple clinicopathological features of gastric cancer, including tumor differentiation, TNM staging (127, 128), and others. This suggests that TREM2 could serve as a potential biomarker for assessing poor prognosis in patients with gastric cancer. Mechanistic studies have shown that TREM2 promotes epithelial-mesenchymal transition (EMT) by activating the PI3K/AKT signaling pathway (127). In colorectal cancer (CRC) (47) and pancreatic cancer (129), high TREM2 expression was significantly negatively correlated with OS and RFS in patients, thus suggesting its potential oncogenic role.

As for renal cell carcinoma (RCC), research has shown that the expression level of TREM2 in RCC tumor tissues was abnormally elevated, and that TREM2 exerts a crucial oncogenic effect by modulating apoptosis-related proteins and the PTEN-PI3K/AKT signaling pathway (130). Additionally, transcriptome sequencing (ST-seq) analysis revealed a co-expression pattern of TREM2 and TREM1 in the tumor-infiltrating myeloid cells of high-grade clear cell renal cell carcinoma (HG ccRCC) (131). Among them, the C1Q^+^ TREM2^+^ APOE^+^ macrophage subset was found to be closely related to the progression and recurrence of RCC (131, 132). Additionally, in the tumor microenvironment of cribriform prostate cancer, there was found to be an increase in the number of C1QB^+^ TREM2^+^ APOE^+^ macrophages. In metastatic foci, TREM2^+^ macrophages are specifically localized at the invasive edges of metastatic nodules, suggesting that TREM2 may be involved in prostate cancer metastasis (133).

Similarly, studies have shown that the proportion of TREM2^+^ TAMs is significantly higher than that in other types of cancer and exhibits a notable correlation with the disease stage and poor prognosis of patients with ovarian cancer (134). However, in high-grade serous cancer (HGSC) with high infiltration of immune cells, TREM2 may play a key role in regulating the polarization of TAMs towards the M1 phenotype, thereby contributing to the promotion of anti-tumor immune responses (135). In triple-negative breast cancer (TNBC), high TREM2 expression has been closely associated with shortened overall OS and RFS (47). Among a subset of patients with TNBC with a poor response to treatment to paclitaxel combined with the PD-L1 inhibitor atezolizumab, there was a significant increase in the number of TREM2^hi^ macrophages (136). *In vitro* experiments have further confirmed that TREM2^+^ macrophages can inhibit T cell activation and the secretion of IFN-γ, thereby contributing to tumor immune escape (137). Notably, researchers have found TREM2^+^ LAMs at the marginal areas of breast cancer lung metastases, suggesting that TREM2^+^ LAMs may play a crucial role in promoting breast cancer lung metastasis (137–139).

By analyzing scRNA-seq data from tumor samples of melanoma patients treated with immune checkpoint therapy (ICT), combined with multiple publicly available scRNA-seq datasets (including GSE120575, GSE115978, and GSE123813), researchers identified a subset of TREM2^hi^ macrophages characterized by high expression of M2-type macrophage polarization genes. This subset of macrophages promotes resistance to ICT by suppressing anti-tumor immune responses (140). TREM2, a receptor expressed on immune cells, has recently been found to be associated with cell pyroptosis. To further investigate the role of TREM2 in melanoma, researchers established a uveal melanoma (UM) mouse model by subcutaneously injecting the UM cell line C918 into BALB/c-nu nude mice. On the 10th day after tumor inoculation, mice were intraperitoneally administered varying doses of the natural compound piceatannol (PIC) for two weeks. Experimental results demonstrated that PIc activates TREM2, thereby triggering the caspase 3/GSDME signaling pathway and inducing non-classical pyroptosis in melanoma cells, leading to significant inhibition of tumor growth. To validate the critical role of TREM2, researchers knocked down TREM2 expression *in vitro* using RNA interference (RNAi) technology and found that the inhibitory effect of PIC on the proliferation of UM cell lines C918 and Mum-2b was significantly reversed. These results indicate that TREM2 plays a key role in PIC-induced pyroptosis in UM cells (141). Furthermore, based on RNA-seq data analysis of skin cutaneous melanoma (SKCM) patients from the TCGA database, researchers found that TREM2 expression levels were significantly higher in SKCM tissues compared to normal tissues. Further analysis using the TIMER (Tumor Immune Estimation Resource) database revealed that TREM2 is closely associated with the infiltration of various immune cells and favorable prognosis in SKCM patients (142). These findings provide new insights into the biological functions of TREM2 in melanoma and its clinical significance.

Furthermore, in the MCA/1956 sarcoma model, both TREM2 deficiency and anti-TREM2 mAb treatment triggered significant changes in the tumor-infiltrating macrophage population, including a decrease in CX3CR1^+^ MRC1^+^ immunosuppressive macrophages and an increase in CD83^+^ CXCL9^+^ immunostimulatory macrophages, thus enhancing the activation of CD8^+^ T cells. Both approaches exhibit synergistic effects when combined with anti-PD-1 therapy (47). In the context of acute myeloid leukemia (AML), IL-34, through binding to TREM2, induces the phosphorylation of Ras protein activator like 3 (Rasal3), which subsequently inhibits the ERK1/2 signaling pathway, promoting the differentiation of AML cells into mature myeloid cells, thereby arresting the progression of AML (143).

## 6 Targeting TREM2 as a therapeutic strategy

Considering the key role of TREM2 in macrophages, intervention strategies targeting TREM2 may hold significant therapeutic potential for the treatment of immune-related diseases (**Table 3**). The 5xFAD mice carrying the R47H mutation (TREM2 R47H-5xFAD mice) exhibit significant suppression of microglial activation and function in response to Aβ pathology, thereby exacerbating neuroinflammatory responses and cognitive dysfunction. Experimental results demonstrate that administration of a TREM2-activating antibody hT2AB to TREM2 R47H-5xFAD mice effectively restores microglial activation and significantly ameliorates survival deficits in these mice. This finding provides important experimental evidence for the therapeutic potential of TREM2 in neurodegenerative diseases (144). Recently, researchers have innovatively designed a bispecific TREM2 agonist monoclonal Ab (Ab18), that is engineered by configuring a divalent IgG1 into a tetravalent variable domain TVD-Ig and an antibody that targets the mouse α transferrin receptor (αTfR). Ab18 TVD-Ig exhibited a 109-fold increase in TREM2 signaling activation compared to the original Ab18. Furthermore, by leveraging the TfR-mediated transcytosis pathway to facilitate the delivery of antibodies across the blood-brain barrier (BBB), there was a significant enhancement in the concentration and distribution of antibodies within the brain. In 5xFAD mice, treatment with Ab18 TVD-Ig significantly reduced the number and size of Aβ plaques, improved synaptic function and neuronal damage, and enhanced cognitive function in the mice (145). Notably, researchers have screened monoclonal antibodies that target the extracellular domain of TREM2 and discovered that the 4D9 antibody exerts its effects through a dual mechanism: reducing the proteolytic shedding of TREM2 from the cell surface and activating the Syk signaling pathway, thereby enhancing TREM2 function. In APP transgenic mice (APP-NL-G-F knock-in mice) used to model AD pathology, 4D9 treatment significantly increased TREM2 expression on microglia and reduced Aβ plaque burden (146). Ldlr^−/−^ mice, which spontaneously develop atherosclerotic plaques on a high-fat diet (HFD), are used as a model for atherosclerosis. After treatment with the 4D9 antibody, the necrotic core area in atherosclerotic plaques of the aortic sinus in Ldlr^−/−^ mice was significantly reduced (104). These findings indicate that the 4D9 antibody enhances TREM2 function and shows potential therapeutic effects in both Alzheimer’s disease and atherosclerosis. Furthermore, a related study evaluated a mouse TREM2-activating antibody, ATV:4D9, that utilizes antibody transport vehicle (ATV) technology to facilitate its transport across the BBB and its distribution within the brain through the TfR. ATV:4D9 enhances the function of protective microglia in the brain of 5xFAD mice by reducing TREM2 proteolytic shedding and amyloid pathology in AD mouse models. Similar to ATV:4D9, ATV: TREM2, an antibody targeting human TREM2, demonstrated increased microglial activity and cerebral glucose metabolism in the 5×FAD mouse model compared to the isotype control antibody (ATV: ISO) group, suggesting a potentially positive impact on the treatment of Alzheimer’s disease (AD) (147). Building on these findings, Haass partnered with Denali to develop DNL919 (ATV: TREM2) for clinical trials (NCT05450549). Unfortunately, this project was ultimately terminated because of the narrow therapeutic window of ATV: TREM2.

**Table 3:** Targeting TREM2 as a therapeutic strategy.

Drug name	Mechanisms	Conditions	Clinical trial number	Phase	Current Status	Sponsor	References
Ab18 TVD-Ig/TfR	anti-TREM2/TfR bispecific antibody	AD	N/A	N/A	N/A	N/A	(145)
4D9	Stabilization of TREM2	AD	N/A	N/A	N/A	N/A	(104, 146)
ATV: 4D9	ATV-enabled TREM2 agonist	AD	N/A	N/A	N/A	N/A	(147)
DNL919	ATV-enabled TREM2 agonist	Healthy Participant	NCT5450549	I	Completed	Denali Therapeutics Inc.& Takeda	N/A
VGL101	anti-TREM2 mAb agonist	ALSP	NCT05677659	II	Active, not recruiting	Vigil Neuroscience, Inc.	(148)
VG-3927	small-molecule TREM2 agonist	Healthy; AD	NCT06343636	I	Completed	Vigil Neuroscience, Inc.	(149)
AL002	anti-TREM2 mAb agonistic	Healthy; AD	NCT03635047	I	Completed	Alctor Inc.	(150, 151)
AL002	anti-TREM2 mAb agonistic	AD	NCT04592874	II	Completed	Alector Inc.& AbbVie	(151, 152)
AL002	anti-TREM2 mAb agonistic	AD	NCT05744401	II	Active, not recruiting	Alector Inc.& AbbVie	(152)
VHB937	anti-TREM2 mAb agonist	ALS	NCT06643481	II	Recruiting	Novartis Pharmaceuticals	(153)
PY-314	TREM2 targeting mAb	Advanced Solid Tumor	NCT04691375	I	Terminnated	Ikena Oncology	(134, 154)

AD, Alzheimer's Disease.

ALSP, Adult-onset leukoencephalopathy with axonal spheroids and pigmented glia.

ALS, Amyotrophic Lateral Sclerosis.

Two TREM2 agonists developed by Vigil are currently undergoing clinical trials. One example is VGL101 (NCT05677659), also known as iluzanebart, a fully human TREM2 monoclonal antibody agonist used to treat adult-onset leukoencephalopathy with axonal spheroids and pigmented glia (ALSP), a rare and fatal neurodegenerative disease caused by mutations in the CSF1R gene. CSF1R shares downstream signaling pathways with TREM2, and VGL101 compensates for loss of CSF1R function and rescues microglia function by activating TREM2 (148). In preclinical studies, iluzanebart demonstrated subnanomolar potency and selective engagement with TREM2. In the Phase 1 clinical trial, VGL101 exhibited a favorable safety and tolerability profile in healthy volunteers, with no severe adverse events observed even at doses up to 60 mg/kg. It also showed linear and predictable pharmacokinetics, supporting monthly dosing. VGL101 is currently in Phase 2 clinical trials, and its potential in treating ALSP is highly anticipated (148). Another example is VG-3927 (NCT06343636), a novel small-molecule TREM2 agonist capable of crossing the blood-brain barrier and efficiently activating TREM2 downstream signaling pathways to modulate the function of microglia, for the treatment of AD (149). Preclinical studies of VG-3927 have shown that it can elicit TREM2 responses in the central nervous system with a magnitude and specificity similar to that of VGL101. In Phase I clinical trials, VG-3927 achieved a significant and sustained decrease in sTREM2 in the cerebrospinal fluid of healthy volunteers, with an increase in osteopontin/secreted phosphoprotein 1 (SPP1) after repeated dosing.

Additionally, AL002, a humanized monoclonal antibody agonist targeting TREM2 developed by Alector, has demonstrated the ability to effectively penetrate the blood-brain barrier, bind to and activate the TREM2 signaling pathway. This activation promotes the survival, proliferation, and phagocytic function of microglia, potentially slowing the progression of AD. In healthy volunteers, AL002 demonstrated good safety and tolerability and dose-dependently reduced sTREM2 levels in cerebrospinal fluid (CSF). The Phase 1 clinical trial (NCT03635047) has been completed (150). Subsequently, Alector and AbbVie collaborated to advance AL002 into a Phase 2 clinical trial (NCT04592874) (151). However, in November 2024, Alector Therapeutics announced that AL002 failed to meet the primary and secondary endpoints of the INVOKE-2 Phase 2 trial. Moreover, AL002 did not show a significant impact on AD-related liquid biomarkers, and amyloid PET imaging showed no treatment-related reduction in brain amyloid levels. It is worth noting that a long-term extension study of INVOKE-2 is currently underway in a Phase 2 clinical trial (NCT05744401) and is scheduled to be completed by December 2025 (152).

VHB937, developed by Novartis Pharmaceuticals, is a fully humanized monoclonal antibody that selectively activates TREM2 in human M2A macrophages and human induced pluripotent stem cells (iPS cells) with subnanomolar affinity. By reducing shedding, it increases the surface expression of TREM2, leading to enhanced Syk phosphorylation and calcium signaling, thereby potentially slowing the progression of human neurodegenerative diseases. Currently, VHB937 is in Phase 2 clinical trials (NCT06643481), aiming to evaluate its efficacy and safety in patients with early-stage ALS (within 2 years of symptom onset) (153).

The above strategies aim to enhance TREM2 signaling to promote the healing activities of macrophages and microglia. However, in oncology, the anti-inflammatory and immunosuppressive effects of TREM2 may facilitate tumor growth and immune evasion. One effort, led by Ikena Oncology, involved utilizing an anti-TREM2 mAb (PY-314) as a monotherapy and in combination with PD-1 inhibitor pembrolizumab, to assess safety, tolerability, pharmacokinetics, and pharmacodynamics in patients with advanced solid tumors, such as gynecologic cancer, breast cancer, triple negative breast cancer, hormone receptor/growth factor receptor-negative breast cancer, ovarian cancer, colorectal cancer, lung adenocarcinoma and renal cell carcinoma (NCT04691375) (134). A study has shown that the combination of PY314 and pembrolizumab has a favorable safety profile in patients with checkpoint inhibitor (CPI)-refractory metastatic renal cell carcinoma (RCC), but the observed anti-tumor effects were limited, suggesting that targeting TREM2 in combination with PD-1 blockade may not overcome resistance to prior CPI therapy (154). Although TREM2 is associated with immunosuppression and poor prognosis in the RCC tumor microenvironment, the depletion of tumor-associated macrophages by PY314 did not significantly translate into clinical anti-tumor benefits. The clinical trial has now been terminated, further research in broader patient populations is still needed to explore the potential of TREM2-targeted therapies.

## 7 Conclusions and perspectives

TREM2 plays a pivotal role in regulating immune responses and maintaining cellular homeostasis, with effects spanning various cell types and disease contexts. As our understanding of TREM2 deepens, the therapeutic potential of targeting this receptor has garnered increasing attention. This review highlights the significant roles of TREM2 in phagocytosis, lipid metabolism, cell survival, and anti-inflammatory processes, further underscoring its importance in multiple pathological states. In neurodegenerative diseases, particularly, TREM2’s role in amyloid plaque clearance identifies it as a potential therapeutic target. Similarly, TREM2’s involvement in lipid metabolism and inflammation suggests it as a candidate target for high-risk diseases associated with metabolic syndrome. However, the dual role of TREM2 in cancer where it can either inhibit or promote tumor development adds complexity to its potential as a therapeutic target. Although preliminary clinical trials targeting TREM2 are ongoing, further exploration is needed into the immune mechanisms underlying the effects of TREM2 blockade therapies. Currently, many questions remain regarding how TREM2 influences the phenotype and function of TAMs and the potential role of sTREM2 in the tumor microenvironment. Future research should focus on investigating the complex signaling networks involved TREM2 and their interactions with other immune modulators. A deeper understanding of these interactions is essential for designing rational therapies that can activate or inhibit TREM2 signaling in specific contexts.
